# Simultaneous targeting of androgen receptor (AR) and MAPK-interacting kinases (MNKs) by novel retinamides inhibits growth of human prostate cancer cell lines

**DOI:** 10.18632/oncotarget.3084

**Published:** 2014-12-26

**Authors:** Vidya P. Ramamurthy, Senthilmurugan Ramalingam, Lalji Gediya, Andrew K. Kwegyir-Afful, Vincent C.O. Njar

**Affiliations:** ^1^ Department of Pharmacology, University of Maryland School of Medicine, Baltimore, MD, USA; ^2^ Center for Biomolecular Therapeutics, University of Maryland School of Medicine, Baltimore, MD, USA; ^3^ Marlene Stewart Greenebaum Cancer Center, University of Maryland School of Medicine, Baltimore, MD, USA

**Keywords:** androgen receptor, eIF4E, MNK, novel retinamides, prostate cancer

## Abstract

Androgen receptor (AR) and MNK activated eIF4E signaling promotes the development and progression of prostate cancer (PCa). In this study, we report that our *Novel Retinamides* (NRs) target both AR signaling and eIF4E translation in androgen sensitive and castration resistant PCa cells via enhancing AR and MNK degradation through ubiquitin-proteasome pathway. Dual blockade of AR and MNK initiated eIF4E activation by NRs in turn induced cell cycle arrest, apoptosis, and inhibited cell proliferation. NRs also inhibited cell migration and invasion in metastatic cells. Importantly, the inhibitory effects of NRs on AR signaling, eIF4E translation initiation and subsequent oncogenic program were more potent than that observed with clinically relevant retinoids, established MNK inhibitors, and the FDA approved PCa drugs. Our findings provide the first preclinical evidence that simultaneous inhibition of AR and eIF4E activation is a novel and efficacious therapeutic approach for PCa, and that NRs hold significant promise for treatment of advanced prostate cancer.

## INTRODUCTION

Androgen receptor (AR), a ligand dependent transcription factor plays pivotal role in the development and progression of prostate cancer (PCa), the most frequently diagnosed non-cutaneous male malignancy [1,2]. While majority of prostate cancers are initially androgen dependent and respond to androgen ablation therapy, most patients eventually recur with more aggressive castration-resistant prostate cancer (CRPC) where AR signaling is reactivated even in the absence of androgen stimulation [2,3].

Besides AR signaling, hyper-activation of eukaryotic translation initiation factor 4E (eIF4E), the mRNA 5′ cap-binding protein of cap dependent translation promotes exquisite transcript-specific translation of key mRNAs that are indispensable in PCa initiation, progression and metastases [4]. The oncogenic potential of eIF4E is dependent on serine 209 phosphorylation by MAPK-interacting kinases 1/2 (MNK1/2) [5]. Overexpression of MNK and hyper-activation of eIF4E are common in hormone-refractory and metastatic prostate tumors and correlates with poor clinical outcome [6-10]. Analysis of the molecular networks in androgen dependent and independent PCa has revealed direct targeting of the translational machinery, precisely eIF4E to be beneficial in the treatment of CRPC [11]. Thus, a strong rationale exists for identifying new drugs that can simultaneously target both AR and eIF4E for treating advanced PCa.

Retinoic acid metabolism blocking agents (RAMBA), a family of compounds that inhibit the P450 enzyme(s) responsible for the metabolism of all-*trans*-retinoic acid (ATRA) exert potent anticancer and growth inhibitory effects in human breast/prostate cancer cells and xenograft models [12-18]. Series of RAMBA related compounds referred to as “Novel Retinamides” (NRs) which are novel structural analogues of ATRA and 4-hydroxyphenyl retinamide (4-HPR) have been designed and synthesized in our laboratory [12-18]. Recently, we demonstrated that these NRs inhibit the growth of triple negative, estrogen receptor alpha (ER-α) and Her2-overexpressing breast cancer cells by blocking MNK initiated eIF4E activation [19].

In the current study, we explored the effect of NRs to simultaneously target AR signaling and MNK activated eIF4E translation initiation in androgen sensitive and CRPC cells. Our data reveal that NRs are capable of inhibiting the growth and progression of PCa by directly targeting both AR signaling and eIF4E translational machinery via enhancing AR and MNK degradation through the ubiquitin-proteasome pathway, which in turn led to inhibition of downstream events that promote cell growth, proliferation, colony formation, apoptosis evasion, invasion and metastasis. Our findings establish for the first time that agents such as NRs, which simultaneously inhibit activation of both AR and eIF4E to suppress growth and progression in genetically diverse PCa cells at pharmacologically feasible concentration, are novel therapeutics for treatment of both androgen-sensitive and castration resistant PCa.

## RESULTS

### Novel retinamides inhibit growth and colony formation in PCa cells

**Figure 1:**
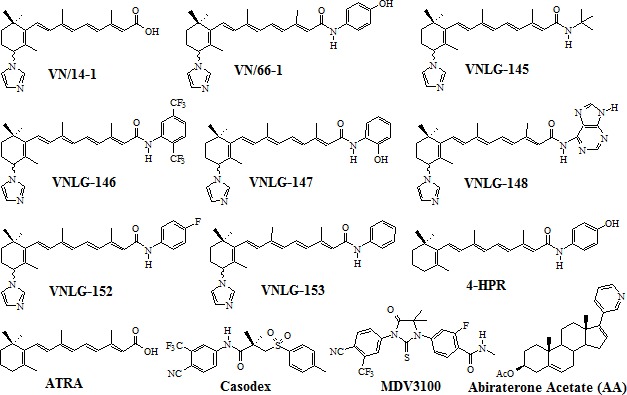
Chemical structures of compounds

**Figure 2:**
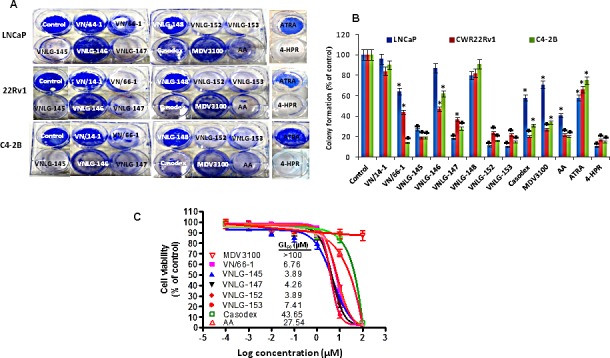
Effect of NRs on PCa cell growth and colony formation (A) Representative photographs of colonies formed in PCa cells at 14^th^ day after initial treatment with specified compounds (5 μmol/L). Colonies were fixed with methanol and stained with crystal violet. (B) Data represents the mean ± S.E from three independent experiments. *, *P*< 0.05; ♣, *P*<0.01 compared to vehicle treated control. (C) Effect of NRs on growth of Enzalutamide resistant MR49F cells. NRs that had promising inhibitory effect on cell proliferation on LNCaP cells were tested for their growth inhibitory effect in MR49F cells in comparison with Casodex and Abiraterone acetate by MTT assay.

To determine the effect of NRs on PCa cell growth, androgen-sensitive (LNCaP) and -insensitive (PC-3, C4-2B) and castration-resistant (22Rv1) human prostate cancer cells were treated with NRs (VN/66-1, VNLG-145, -146, -147, -148, -152, -153), VN/14-1, ATRA, 4-HPR, Casodex, Abiraterone acetate (AA) (Zytiga) and MDV3100 (Enzalutamide) (Figure 1) and assessed for cell viability by MTT assay. We found that NRs, particularly VNLG-145, -147, -152 and -153 inhibited the growth of PCa cells with a GI_50_ value 1.5 - 5.5 μmol/L (Table 1, Supplementary Figure 1 and 2). The growth inhibitory potential of these NRs were more potent than that observed for VN/66-1, our previously established lead RAMBA; VN/14-1, the parent RAMBA from which the new NR series (VNLG-145, -146, -147, -148, -152, -153) were derived; clinically relevant retinoids (ATRA and 4-HPR) and almost comparable to that of the FDA approved PCa drugs (Casodex, AA, MDV3100). Our results also indicated that the growth inhibitory effect of NRs in PCa cells was significantly much higher (12-30 fold) compared to that in immortalized untransformed prostate cells signifying that NRs are differentially sensitive towards untransformed and malignant prostate cells. The other NRs (VNLG-146 and -148) did not display any notable growth inhibitory effect or differential sensitivity (Table 1). Analysis of the effect of NRs on colony forming ability of PCa cells, indicated that lead NRs in addition to inhibiting cell viability were also able to inhibit PCa colony formation (Figure 2A and B).

We next evaluated the growth inhibitory potential of NRs, specifically VN/66-1, VNLG-145, -147, -152, -153 that showed promising growth and colonization inhibitory potential in prostate cancer cells in comparison with casodex, AA and MDV3100 in LNCaP cells that were resistant to MDV3100 treatment (MR49F). Our results revealed that NRs significantly inhibited the growth of these resistant cells with a GI_50_ value ≅ 3.5 – 7.5 μmol/L, which was several times more potent than that observed for the FDA approved drugs Casodex or AA (GI_50_: 43.65 and 27.54 μmol/L respectively). We also note that Enzalutamide had no significant effect up to a concentration of 100 μmol/L (Figure 2C).

**Table 1:** Antiproliferative potencies of NRs, ATRA, 4-HPR, and FDA approved PCa drugs in androgen sensitive and castration resistant human prostate cancer cell lines

Compound	PWR-1E (μM)	PC-3 (μM)	C4-2B (μM)	22Rv1 (μM)	LNCaP (μM)	LNCaP (μM, DHT induced)
VN/14-1	69.18	36.3	9.76	12.88	11.32	12.88
VN/66-1	19.05	10.71	2.38	2.95	4.67	1.54
VNLG-145	25.11	4.26	2.14	2.23	2.45	2.23
VNLG-146	63.09	57.54	11.74	39.81	57.54	52.48
VNLG-147	71.53	2.95	2.66	2.04	1.86	1.41
VNLG-148	43.65	36.3	15.48	39.81	22.9	19.05
VNLG-152	47.86	5.62	1.54	3.23	2.45	1.86
VNLG-153	39.81	4.36	2.97	2.23	3.23	2.69
ATRA	75.3	36.3	11.74	25.11	47.86	39.81
4-HPR	1.07	3.54	3.11	3.23	2.69	2.45
Casodex	69.1	9.15	1.51	3.81	2.61	2.95
MDV3100	16.98	9.15	1.54	3.34	2.88	2.69
AA	20.15	7.62	1.47	2.97	2.45	2.69

Note: Cells were treated with listed compound (0.1 nmol/L-100 μmol/L) for 7 d and the GI_50_ values for the antiproliferative effects of the compounds were determined from dose response curves (by a nonlinear regression analysis using Graph Pad Prism). Data represents the results from six independent experiments for each cell line.

### NRs modulate androgen receptor (AR) expression and activity in cultured human prostate cancer cells

**Figure 3:**
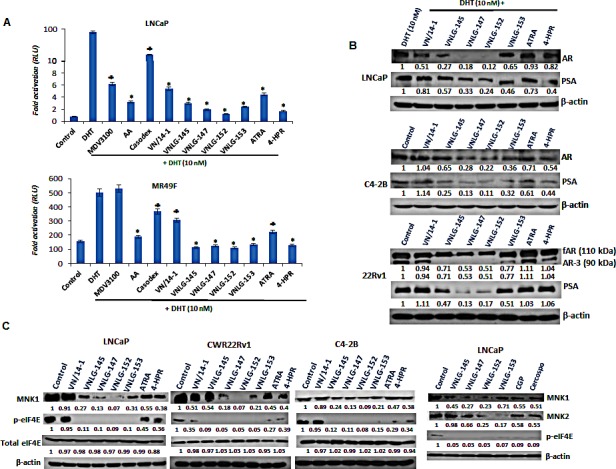
Effects of NRs on AR, MNK and eIF4E in PCa cells (A) LNCaP and MR49F cells dual transfected with ARR2-Luc and the *Renilla* luciferase reporting vector were tested by luciferase assay following treatment with 10 μM of specified compounds for 24 h. *, P< 0.01; ♣, P<0.05 compared with DHT treated cells. (B) Western blotting for fAR, AR3 and PSA. Cells were treated with indicated compound at concentration of 20 μmol/L for 24 h. Total cell lysates were separated by SDS-PAGE and probed with specific antibodies. Vehicle treated cells were included as a control and all blots were reprobed for β-actin for equal protein loading and transfer. (C) Western blots representing MNK 1/2, eIF4E and peIF4E in PCa cells.

Since AR is a major driver of proliferation in PCa [20], we next examined the effect of NRs on AR transcriptional activity in PCa cells. As shown in Figure 3A, 24 h exposure of LNCaP cells to lead NRs (10 μM) resulted in a 2 - 4 fold dramatic inhibition of DHT induced AR transcriptional activity that was far more potent than that observed upon ATRA, 4-HPR, Casodex, MDV3100 or AA treatments. We further examined the effect of NRs in inhibiting AR transcriptional activity in Enzalutamide resistant cells. Our results indicated that lead NRs at a concentration of 10 μM resulted in a signiﬁcant decrease in DHT induced AR transcriptional activity whereas Enzalutamide had no effect. The observed decrease in DHT induced AR transcriptional activity of lead NRs was comparable to that seen in AA treated cells and stronger than that detected upon Casodex treatment (Figure 3A).

To further determine whether the inhibition of transcriptional activity could be translated to inhibition of protein expression, we next explored the effects of NRs on AR and its responsive protein, PSA in DHT induced LNCaP cells. As seen in Figure 3B, 24 h treatment of LNCaP cells with lead NRs caused a signiﬁcant down-regulation in the expression of both full-length AR (fAR) and its target gene, PSA. A similar pattern of result was observed upon NRs treatment in C4-2B and 22Rv1 cells. In addition, in 22Rv1 cells lead NRs were also able to down-regulate the expression of AR splice variant AR3 (Figure 3B). The down-regulatory effects of NRs on fAR, AR3 and PSA were more potent than that observed with ATRA and 4-HPR in all the PCa cells analyzed.

### NRs simultaneously reduce MNK and peIF4E expression in PCa cells

We next examined the expression of MNK and eIF4E (total and Ser^209^ phosphorylated form) in three PCa cell lines in comparison with established retinoids and known MNK inhibitors, CGP57380 and cercosporamide. We observed that 24 h treatment of PCa cells with lead NRs reduced the expression of MNK1, MNK2 and peIF4E^ser209^ with no notable effect was on the expression of total eIF4E (Figure 3C). The observed decrease in the expression of MNKs and peIF4E^ser209^ were more pronounced than that observed upon treatment with ATRA, 4-HPR, and MNK inhibitors.

### NRs inhibited prostate cancer cell growth, cell migration and invasion, and induced cell apoptosis

**Figure 4:**
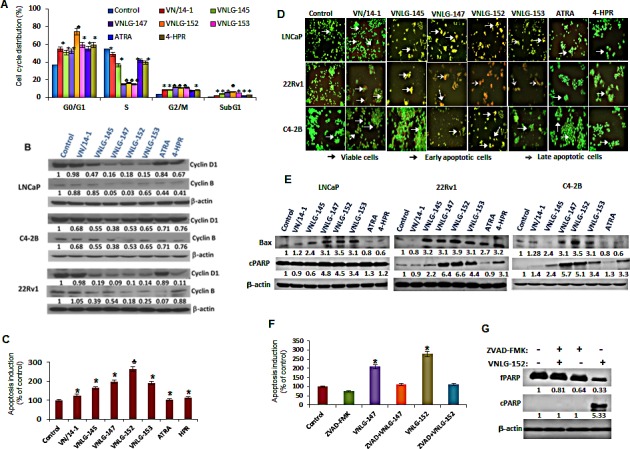
Effect of NRs on cell cycle and apoptosis (A) LNCaP cells treated with 5 μM of NRs and other compounds for 24 h were stained with PI and analysed with a FACS calibur flow cytometer. (B) Total cell lysates from PCa cells treated with 20 μM of NRs were separated by SDS-PAGE and probed with cyclin D1 and B antibodies. Vehicle treated cells were included as a control and all blots were reprobed for β-actin for equal protein loading and transfer. (C) LNCaP cells were treated with indicated compounds (5 μmol/L) for 24 h and apoptosis induction was examined by oligonucleosomal fragmentation. Data are shown relative to vehicle treated control and the bars are means of three replicate determinations plus standard deviations. *, *P*< 0.01; ♣, *P*<0.05 compared with vehicle treated control. (D) PCa cells as indicated were seeded in 24 well plate, and treated with 5 μmol/L of NRs the subsequent day. After 24 h the plates were analyzed for apoptotic and viable cells using acridine orange/ethidium bromide staining. (E) Western blot analysis of apoptosis associated Bax and cPARP in PCa cells treated with NRs (20 μmol/L) for 24 h. (F) Apoptosis induction in LNCaP cells treated with 5 μmol/L of VNLG-147 and -152 in the presence or absence of ZVAD (5 μmol/L) was assessed by oligonucleosomal fragmentation after 24 h incubation. *, *P*< 0.05 compared with vehicle treated control. (G) Expression of full-length and cleaved PARP protein was investigated by Western blot in LNCaP cells treated with lead NR (VNLG-152, 20 μM) with or without caspase inhibitor ZVAD (5 μmol/L).

We next sought to determine the functional relevance of AR and MNK/peIF4E downregulation on cell cycle and apoptosis- the major downstream effect of constitutive AR signaling and eIF4E activation in malignant PCa cells. As shown in Figure 4A, 24 h treatment of PCa cells with VN/14-1, VNLG-145, -147, -152 and -153 (5 μM) reduced the number of cells in S phase and concomitantly increased their population in G2/M phase (8.89, 8.43, 11.6, 11.0, 11.2, 7.5 and 8.3 % respectively) compared to untreated cells (4.05%). In VNLG-152 treated LNCaP cells, in addition to an increase in G2/M phase cells there was also a remarkable increase in the percentage of cells in G1 phase (74.6%) compared to untreated control (37.1%). NRs induced cell cycle arrest was also accompanied by simultaneous decrease in the expression of cyclins D1 and B that are associated with G1/S and M cell cycle phases (Figure 4B).

We next assessed the effect of lead NRs on apoptosis in PCa cells by acridine orange-ethidium bromide dual staining. As seen in Figure 4D, viable cells stained only by acridine orange were bright green with intact structure, whereas apoptotic cells (induced by NR treatment) stained predominantly by ethidium bromide and to a slight extent by acridine orange were orange-red colored displaying cell shrinkage, chromosomal condensation, and nuclear fragmentation, the characteristic features of apoptosis [21]. The apoptosis inducing potential of NRs was also confirmed quantitatively by ELISA assay (Figure 4C). Analysis of the expression of apoptosis associated proteins, Bax and PARP-1 cleavage by Western blot analysis revealed an increase in the expression of pro-apoptotic Bax and cleaved PARP-1 in NRs (20 μM) treated PCa cells relative to control. Among the lead NRs, VNLG-147 and -152 exhibited greater efficacies in inducing PARP cleavage and/or increasing Bax expression (Figure 4E). Additionally, LNCaP cells were treated with VNLG-147/-152 in the presence or absence of pan-caspase inhibitor ZVAD (5 μM, 24 h) to confirm whether apoptosis induction by NRs was caspase mediated. Our results showed that combined treatment of ZVAD completely suppressed NRs induced oligonucleosomal fragmentation (Figure 4F) and PARP-1 cleavage (Figure 4G) confirming that NRs induced apoptosis via the caspase-dependent pathway.

**Figure 5:**
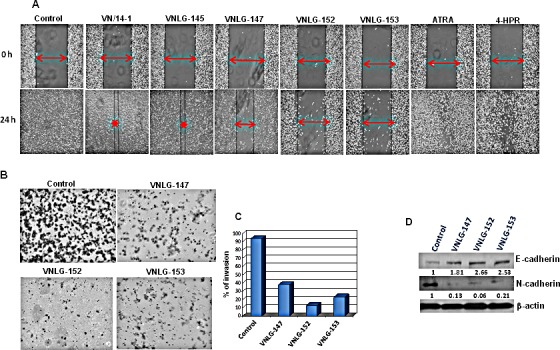
Anti-migratory and anti-invasive potential of NRs (A) Effect of NRs treatment (5 μmol/L) on PC-3 cell migration was assessed by wound healing assay after 24 h of wound formation. Representative photomicrographs of initial and final wounds are shown at 100x magnification. (B) Effect of NRs on PC-3 cell invasion was evaluated by transwell migration assay. PC-3 cells were seeded on matrigel coated boyden chamber and treated with NRs (5 μmol/L, 24 h). Photographs represent the extent of cell invasion in each of the treated cells. (C) Western blot analysis of the effect of NRs on the expression of cadherins in PC-3 cells. Cells were treated with indicated compound 20 μmol/L for 24 h. Total cell lysates were separated by SDS-PAGE and probed with E- and N-cadherin antibodies. Vehicle treated cells were included as a control and all blots were reprobed for β-actin for equal protein loading and transfer.

We also evaluated the inhibitory effects of NRs on cell invasion and migration- the late stage carcinogenic events in the metastatic PCa cell line, PC-3 by wound-healing and trans-well invasion assays [22]. We found that 24 h after cell monolayers were wounded control cells had completely filled in the scratched area. Whereas incubation of PC-3 cells with NRs, in particular VNLG-147, -152 and -153 at concentration of 5 μM for 24 h potentially suppressed PC-3 cell migration to the denuded zone (Figure 5A), revealing that NRs potentially inhibit the motility of PC-3 cells. To further elucidate the inhibitory effect of NRs on the invasion of PC-3 cells across extracellular matrix (ECM), the cells that invaded through the matrigel coated polycarbonate filter in the Boyden chamber were analyzed. Our results showed that NRs treatment (5 μM) for 24 h profoundly suppressed invasion of PC-3 cells across the matrigel-coated filter. The percentage of cell invasion was 33, 13 and 35 for VNLG-147, -152 and -153, respectively compared to control (Figure 5B and C). In addition, lead NRs were also able to modulate the expression of E- and N-cadherin, the key players in epithelial mesenchymal transition (EMT) (Figure 5D). Our results thus signify that NRs induced anti-migratory and anti-invasive effects in PC-3 cells is primarily due to their modulatory effects on cell adhesive cadherins.

These data collectively suggest that NRs exhibit potent anti-cancer effects that are more pronounced than parent RAMBA, VN/14-1 and the clinically relevant retinoids ATRA and 4-HPR. Amongst the NRs, VNLG-152 exhibited the most impressive anti-cancer effects and was therefore chosen for further detailed investigations.

### AR and MNK are the prime targets of VNLG-152's anti-cancer effects in PCa cells

**Figure 6:**
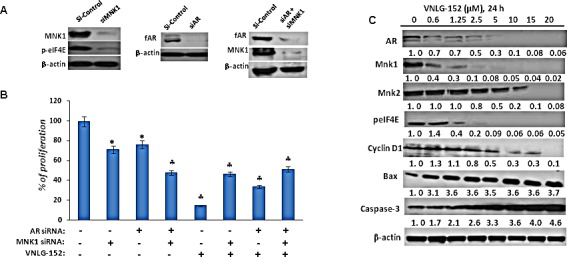
Effect of siAR, siMNK1 on LNCaP cell viability, and VNLG-152 on key and downstream proteins in LNCaP cells (A) Western blot analysis of the expression of fAR, MNK1 and peIF4E in LNCaP cells transfected with 100 nM of siAR, siMNK1 and combinations for 18 h. (B) Effect of VNLG-152 (5 μmol) on cell proliferation in LNCaP cells transfected with siAR, siMNK1 and combinations as determined by MTT assay. The results represent the mean ± SEM of three independent experiments and are represented as a bar graph after normalizing to control cells. *, *P*< 0.05; ♣, *P*<0.01 compared with vehicle treated control. (C) Dose-dependent effect of VNLG-152 on the expression of AR, MNKs, peIF4E and downstream target proteins. Total cell lysate from LNCaP cells treated with VNLG-152 (0 - 20 μmol/L) for 24 h were separated by SDS-PAGE and probed with corresponding antibodies. Vehicle treated cells were included as control and all blots were reprobed for β-actin to ensure equal protein loading.

To confirm whether the observed anti-cancer effects of VNLG-152 on PCa cells was primarily due to its inhibitory effects on AR and MNK, we investigated the impact of AR and/or MNK siRNA on the survival of LNCaP cells by MTT assay. The efficiency of transfection was confirmed by western blot analysis, wherein protein lysates obtained from the transfected cells after AR siRNA transfection showed a temporal decrease in total AR protein and cells transfected with MNK1 siRNA showed a temporal decrease in the expression of both MNK1 and peIF4E compared to scrambled (siSCR) treated controls after 18 h of transfection. Cells co-treated with AR and MNK1 siRNA showed a remarkable decrease in the expression of both AR and MNK1 compared to scrambled siRNA treated cells (Figure 6A). MTT assay revealed that transient transfections with AR or MNK1 siRNA alone for 18 h caused a considerable decrease (≈ 30%) in LNCaP cell viability compared to control (compare lane 1 to lanes 2 and 3). Co-transfection of LNCaP cells with both AR and MNK1 siRNA (compare lanes 1 and 4) further augmented loss of viability in LNCaP cells. Treatment of LNCaP cells (untransfected) with VNLG-152 (5 μM) for 72 h showed a robust reduction in cell viability (lane 5). LNCaP cells harboring MNK1 (lane 6) or AR (lane 7) knockdown also displayed a significant decrease in cell viability upon treatment with VNLG-152 compared to the MNK1 and AR siRNA alone treated counterparts (lane 2 and 3, respectively). However, LNCaP cells with double knockdown of AR and MNK1 genes (lane 8) did not show any significant decrease in cell viability upon VNLG-152 treatment compared to lane 4, which was co-treated with MNK1 and AR siRNA (Figure 6B). These results strongly suggest that MNK1 and AR are indispensable for VNLG-152 to mediate its growth inhibitory effects in LNCaP cells, and that loss of both prime targets abolish the potent growth-inhibitory effects mediated by VNLG-152.

To further authenticate that VNLG-152 induced loss of cell viability at GI_50_ concentration was due to downregulation of AR and MNKs, we performed a dose-dependent analysis by treating LNCaP cells with different concentrations of VNLG-152 (0.6, 1.25, 2.5, 5, 10, 15 and 20 μM) for 24 h. As seen in Figure 6C, VNLG-152 triggered a dose-dependent decrease in the expressions of fAR, MNK1, and peIF4E with notable effect at 2.5 μM concentration (observed GI_50_ concentration of VNLG-152 in LNCaP cells) and above. However, in the case of MNK2, though VNLG-152 exerted a dose-dependent decline maximal effect was observed only at 20 μM concentration. Besides MNKs and AR, a dose-dependent decrease in the expression of cell cycle regulatory cyclin D1 and increase in the expression of pro-apoptotic Bax and caspase was also observed in LNCaP cells at the GI_50_ concentration. These results endorse the fact that VNLG-152 induced loss of cell viability is due to downregulation of AR and MNKs.

### AR and MNK proteins are decreased post-translationally upon VNLG-152 treatment

**Figure 7:**
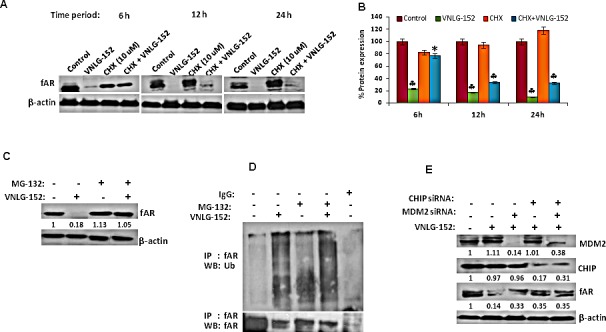
VNLG-152 induced AR degradation by ubiquitin proteasomal pathway in LNCaP cells (A) Western blot analysis of the expression of fAR in LNCaP cells treated with VNLG-152 (20 μmol/L) in the presence or absence of CHX (10 μmol/L) at 6, 12 and 24 h of treatment. (B) Densitometric analysis of the expression of fAR in different groups *, *P*< 0.05; ♣, *P*<0.01 compared with vehicle treated control. (C) Western blot analysis of the expression of fAR in LNCaP cells treated with VNLG-152 (20 μmol/L) in the presence or absence of MG-132 (5 μmol/L). (D) LNCaP cells were treated with 20 μmol/L of VNLG-152, 5 μmol/L of MG-132, and combinations for 24 h. AR protein was immunoprecipitated with AR antibody (mouse) and the precipitated protein was subjected to western blot analysis with anti-ubiquitin antibody (Ub) (C, upper panel). The same blot was used to detect AR protein with anti-AR (rabbit) antibody after stripping (C, lower panel). (E) Total cell lysate from LNCaP cells treated with VNLG-152 (20 μmol/L) in the presence or absence of MDM2 and/or CHIP siRNA were separated by SDS-PAGE and probed with antibodies for fAR, MDM2 and CHIP. Vehicle treated cells were included as control and all blots were reprobed for β-actin to ensure equal protein loading.

**Figure 8:**
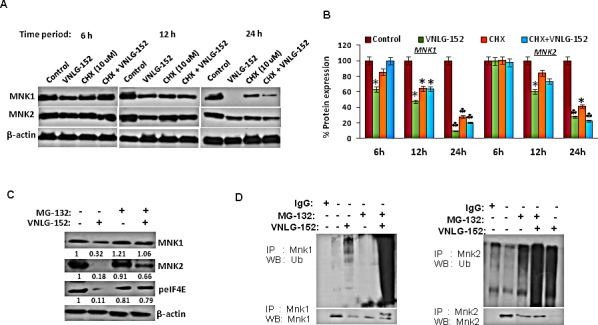
VNLG-152 induced MNK1/2 degradation by ubiquitin proteasomal pathway in LNCaP cells (A) Western blot analysis of the expression of MNK1/2 in LNCaP cells treated with VNLG-152 (20 μmol/L) in the presence or absence of CHX (10 μmol/L) at 6, 12 and 24 h of treatment. (B) Densitometric analysis of the expression of MNK1/2 in different groups *, *P*< 0.05; ♣, *P*<0.01 compared with vehicle treated control. (C) Western blot analysis of the expression of MNK1/2 and peIF4E in LNCaP cells treated with VNLG-152 (20 μmol/L) in the presence or absence of MG-132 (5 μmol/L). Vehicle treated cells were included as control and all blots were reprobed for β-actin to ensure equal protein loading. (D) LNCaP cells were treated with 20 μmol/L of VNLG-152, 5 μmol/L of MG-132, and combinations for 24 h. MNK1/2 protein was immunoprecipitated with MNK1/2 antibody (mouse) respectively and the precipitated protein was subjected to western blot analysis with anti-ubiquitin antibody (Ub) (upper panel). The same blot was used to detect MNK1/2 protein with anti-MNK1/2 (rabbit) antibody after stripping (lower panel).

Since AR and MNK protein levels were signiﬁcantly reduced in response to 24 h treatment of VNLG-152 in a dose-dependent manner, we next investigated the expression of AR and MNK in LNCaP cells following treatment with cycloheximide (CHX), a protein synthesis inhibitor to unveil whether VNLG-152 induced AR and MNK downregulation occurs at the level of protein translation. Our results showed that AR and MNK (MNK1 and MNK2) were profoundly reduced even within 12 h of VNLG-152 treatment compared to control. However, CHX treatment failed to induce noticeable AR/MNK downregulation at the same time points relative to VNLG-152 treated cells signifying that post-translational mechanisms are in play in VNLG-152 induced AR/MNK downregulation (Figure 7A, 7B, 8A and 8B).

### VNLG-152 reduce AR and MNK expression via proteasomal degradation

Because our results revealed that AR and MNK protein ablation by VNLG-152 is post-translational, we asked whether this occurred via ubiquitin–proteasome system. For this we treated LNCaP cells with 20 μM VNLG-152 in the presence or absence of MG-132 (5 μM), the 26S proteasome inhibitor. As seen in Figures 7C and 8C, MG-132 treatment largely restored the AR, MNK (MNK1 and 2) and peIF4E level decreased by VNLG-152. Since inhibition of proteasomal activity by MG-132 leads to an increase in polyubiquitinated form of AR and MNK (Figure 7D and 8D), we stripped and reprobed the same blot with anti-ubiquitin antibody. There was an increase in the extent of ubiquitinated protein in the lanes treated with MG-132 with or without VNLG-152 treatment. This result indirectly shows that MG-132 treatment resulted in accumulation of ubiquitinated form of AR/MNK as proteasomal activity is inhibited; suggesting that VNLG-152 mediated AR/MNK degradation is largely proteasome dependent.

### VNLG-152 induced proteasomal degradation of AR is mediated by E3 ubiquitin ligases (MDM2/CHIP)

Proteasomal degradation of the AR is known to be mediated by the E3 ligases, MDM2 or CHIP [23]. To test the involvement of these E3 ubiquitin ligases in VNLG-152 induced AR degradation, we interfered with AR degradation in LNCaP cells by knocking down CHIP and/or MDM2 using MDM2/ CHIP siRNA. We found that depletion of MDM2 or CHIP signiﬁcantly reduced VNLG-152-induced AR degradation in LNCaP cells (Figure 7E) compared to lane 1 and 2 that represent control and VNLG-152 alone treated cells. We also observed an additional protection of the AR when we knocked down CHIP along with MDM2 (lane 5) suggesting that both CHIP and MDM2 are involved in VNLG-152 induced AR degradation.

## DISCUSSION

Since the importance of AR signaling and MNK mediated eIF4E activation in PCa development and progression has been reported, several strategies to target the respective signaling pathways have been developed [3,20,24,25]. However, simultaneous targeting of AR signaling and MNK facilitated eIF4E activation- the major signaling pathways involved in PCa development, progression, and transition from androgen dependence to androgen resistant form, to develop proof-of-concept small molecule inhibitors as potential PCa therapeutics has not yet been shown [9,20]. Here, we demonstrate for the first time that our proprietary novel retinamides can simultaneously inhibit both AR signaling and MNK facilitated eIF4E translation initiation and impede growth and progression of in androgen dependent (LNCaP) and castration resistant (C4-2B, 22Rv1) prostate cancer cells. Most interestingly, NRs also block AR expression, activity and cell growth in cells that are resistant to enzalutamide treatment. Our data collectively suggest that NRs may be promising novel therapeutics for patients with advanced prostate cancer and those resistant to enzalutamide treatment.

Androgen-AR signaling has a critical role in prostate cancer development and progression in part through transcriptional regulation of AR responsive genes [20]. Earlier studies have validated that AR splice variants, especially the most dominant variant AR3 which lacks a portion of ligand binding domain contributes to the transition of prostate cancer from androgen dependent to castration resistance. While constitutively active AR3 might itself facilitate PCa cell growth and progression, it may even depend on full-length AR to execute AR transcriptional activity and cell growth effects [26]. In the present study, NRs treatment downregulated the expression of full-length AR (in LNCaP, C4-2B and 22Rv1); splice variant AR3 (in 22Rv1); and the AR responsive protein, PSA in genetically diverse PCa cells. Furthermore, NRs also dramatically reduced DHT induced AR transcriptional activation in LNCaP and MR49F, the enzalutamide resistant LNCaP cells. In mechanistic studies identifying lead NR (VNLG-152) mediated AR down-regulation in LNCaP cells, treatment with protein synthesis inhibitor did not have any effect on VNLG-152 induced AR down-regulation, whereas the proteasome inhibitor MG-132 largely restored VNLG-152 induced AR decrease. Knockdown of the E3 ubiquitin ligases, CHIP or MDM2 individually or in combination abrogated VNLG-152 mediated fAR degradation, suggesting that NR (VNLG-152) mediated AR down-regulation in LNCaP cells involved degradation of the active AR protein via proteasomal degradation through the activation of E3 ubiquitin ligases CHIP and MDM2. Our finding that AR is degraded through CHIP is consistent with the degradation of nuclear receptors where ubiquitination has been demonstrated to signal receptor degradation [27]. Involvement of MDM2 in promoting AR ubiquitination and degradation has also been reported in a number of studies [27-29]. Lin *et al*. demonstrated that AR ubiquitination is impaired in MDM2-deficient mouse embryonic fibroblasts (MEFs) compared to MDM2-intact MEFs. Although our work confirms a role for MDM2 and CHIP in degrading AR, the primary E3 ligase responsible for degrading AR in response to VNLG-152 and the molecular mechanisms underlying their preference are not fully understood.

In addition to AR signaling, the role of MNK mediated cap dependent translation in PCa development and progression has been established in recent years [24,30]. Initiation of cap dependent translation primarily involves the assembly of eIF4F initiation complex comprising of eIF4E, eIF4G and eIF4A on the 5′ cap of mRNA [30]. Of all the members of the translation initiation complex, eIF4E expression and activity is considered crucial as this is the only protein that binds directly to the mRNA cap structure [30]. Indeed, overexpression of eIF4E alone has been shown to contribute directly to cellular transformation [4,31]. The transforming properties of eIF4E have been linked to its ability to promote translation of genes involved in proliferation and survival including that of c-Myc, cyclin D1, growth factors (fibroblast growth factor 2, FGF2 and vascular endothelial growth factor, VEGF), and Mcl-1 [30]. The oncogenic potential of eIF4E is sternly dependent on phosphorylation at serine 209 residue by MNK1/2 which increase the affinity of eIF4E for 5′ cap of mRNA.4 Both MNK and p-eIF4E are found to be up-regulated in various cancers [19,32-34]. Bianchini *et al*. demonstrated that eIF4E phosphorylation by MNKs supports protein synthesis, cell cycle progression and proliferation in prostate cancer. Furthermore combined deficiency of MNK1 and 2 has been demonstrated to delay tumour development [35]. In the present study, NRs treatment dramatically reduced the expression of MNK1, MNK2, and peIF4E^ser209^ without affecting total eIF4E expression. Notably, the effect of NRs in depleting MNKs and peIF4E proteins was far potent than the established MNK inhibitors (cercosporamide and CGP57380) and clinically relevant retinoids-ATRA and 4-HPR.

Dissection of the molecular mechanism underlying lead NR (VNLG-152) mediated MNK and peIF4E down-regulation indicated that VNLG-152 stimulated ubiquitination and proteasomal degradation of MNKs, the critical activators of eIF4E. Degradation of MNKs by VNLG-152 may abrogate MNK mediated phosphorylation of eIF4E at serine209, which subsequently impairs its ability to enter the eIF4F initiation complex, binding to 5′ mRNA cap, and activation of cap-dependent translation initiation [30]. The results of the present work are consistent with the earlier report by Ramalingam *et al*. who demonstrated that NRs induce MNK degradation and block eIF4E phosphorylation in triple negative and Her2-overexpressing breast cancers.

Constitutive AR signaling and/or eIF4E mediated translation initiation favors translation of key genes involved in oncogenesis [4,20]. Abrogation of both these pathways may block translation of sub-set of genes indispensable for maintaining growth and survival in PCa cells. In the present study, the functional consequence of AR and MNK knockdown on LNCaP cell proliferation provides proof-of-principle for the importance of AR and MNK mediated signaling in upholding growth and survival of PCa cells, and also authenticates that AR and MNK are the prime target of NR (VNLG-152) for mediating growth inhibitory effects in PCa cells. The biological significance of NRs mediated dual inhibition of AR and MNK mediated signaling pathways is further indicated by enhanced cell death and inhibition of cell growth, cell colonization, migration and invasion in PCa cells. Several studies in the past have elicited a direct role of AR and eIF4E translation initiation in regulating cell cycle progression [19,23,36,37]. Blocking of AR expression has been demonstrated to induce strong cell cycle arrest [37,38]. Results from the present study also show that NRs induce cell cycle arrest at G1/S (VNLG-152) or G2/M (VNLG-145, -147, -153) phase with accompanied decrease in the expression of cyclin B and cyclin D1 that are associated with G1/S and M phase cell cycle transition. Besides inducing cell cycle arrest, NRs treatment also induced caspase dependent apoptosis in PCa cells through increased expression of pro-apoptotic Bax. Our findings indicate that synchronized dual inhibition of AR signaling and MNK mediated eIF4E activation by NRs in PCa cells results in blockade of cell cycle progression and induction of caspase dependent apoptosis which is in line with the earlier reports that demonstrate that targeting AR or MNK in PCa cells results in induction of cell cycle arrest and apoptosis (19,37,38). In the highly metastatic PC-3 cells which are also AR negative, NRs treatment also impaired cell migration and invasion, the late stage events essential for tumour metastasis [39]. The anti-invasive and anti-migratory effects of NRs were associated with reappearance of E-cadherin and down-regulation in the levels of N-cadherin. Earlier, Graff et al. [40] demonstrated that cells with reduced levels of eIF4E had reduced invasiveness and experimental metastasis. Similarly, Li et al. [41] showed that knockdown of eIF4E significantly inhibited invasion of human non-small cell lung cancer (NSCLC) cells. These reports suggest that elevated eIF4E expression is a positive regulator of invasion, and that NRs inhibit migration and invasion, the late stage carcinogenic events in the AR negative PC-3 cells by impeding MNK dependent eIF4E translation initiation that is chiefly responsible for translation of various key genes associated with invasion and metastasis [4,30].

**Figure 9:**
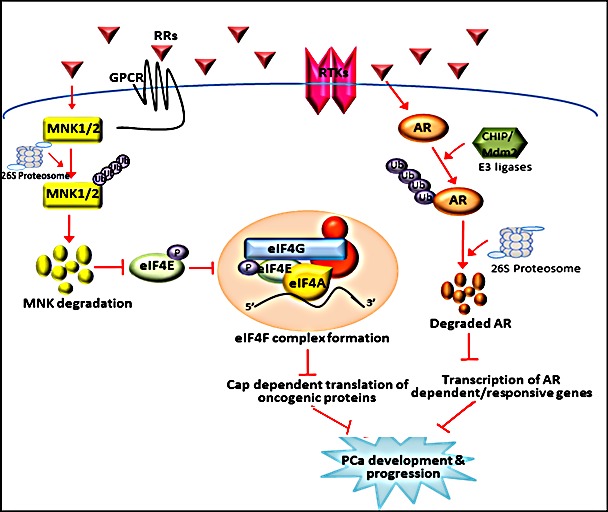
Schematic representation of the inhibition of AR signaling and MNK activated eIF4E cap-dependent translation by NRs NRs stimulate AR degradation in LNCaP cells by promoting its proteasomal degradation through the CHIP or MDM2 E3 ubiquitin ligases. Degradation of AR by in turn obstructs AR mediated signaling and transcription of AR responsive genes that play crucial role in PCa development and progression. Simultaneously NRs can also inhibit MNK activated eIF4E translation initiation in LNCaP cells by promoting proteasomal degradation of MNKs. This event in turn inhibits phosphorylation of eIF4E at ser209 residue, subsequent formation of translation initiation complex and translation of key mRNAs critical for PCa development and progression.

In summary, our study identiﬁes for the first time novel retinamides that can simultaneously inhibit both AR signaling and MNK mediated eIF4E activation in prostate cancer cells (Figure 9). Most intriguingly, our proprietary NRs were able to block AR activation and cell growth in PCa cells resistant to Enzalutamide treatment. NRs also displayed inhibitory and anti-cancer effects in PCa cells in order far more potent than FDA approved PCa drugs, established MNK inhibitors and clinically relevant retinoids, with nominal effects on immortalized untransformed prostate cells. Collectively, these results suggest that novel retinamides that function as dual inhibitors of AR and MNK signaling in androgen-dependent and castration-resistant PCa have great potential as novel therapeutics for treatment of advanced prostate cancer.

## MATERIALS AND METHODS

### Cell culture

Androgen-dependent (LNCaP), androgen-independent (PC-3) and castration resistant (C4-2B and 22Rv1) human prostate carcinoma cells were procured from American Type Culture Collection, Manassas, VA, USA and maintained in RPMI 1640 media (Gibco-Life Technologies, Grand Island, NY, USA) supplemented with 10% fetal bovine serum and 1% penicillin/streptomycin. MR49F, Enzalutamide resistant LNCaP cells (a generous gift of Dr. Amina Zoubeidi, The Vancouver Prostate Centre) were maintained in RPMI 1640 supplemented with 10% fetal bovine serum, 1% penicillin/streptomycin and 10 μM Enzalutamide. Immortalized untransformed prostate epithelial cells, PWR1E from American Type Culture Collection were maintained in serum-free Keratinocyte media (Gibco) supplemented with epidermal growth factor and bovine pituitary extract. All cell lines were maintained in a 37 °C/5% CO_2_ humidified atmosphere.

### Cell proliferation and colony formation assays

Cell proliferation assay was performed as described previously [14]. For cell growth experiment, cells were treated with the NRs for 7 days. MTT assay was performed at the end of the experiment. Calculations of combination indices were done using the Calcusyn program (Biosoft, Cambridge, United Kingdom). For colony formation assay, cells were plated 1000 per well in complete media and allowed to adhere for 24 h. The next day cells were treated with indicated compounds (5 μM). After 24 h compound containing media were removed, and cells were allowed to form colonies in complete media. Approximately 2 weeks later the colonies were fixed, stained with 0.5% crystal violet (sigma) for 30 min and counted manually. Results represent the mean ± standard deviation of three independent experiments.

### Transcriptional activation – luciferase assay

LNCaP cells were transferred to steroid-free medium 3 days before the start of the experiment, and plated at 1 × 10^5^ cells/well in steroid-free medium. The cells were dual transfected with ARR2-Luc and the *Renilla* luciferase reporting vector pRL-null with LipofectAMINE 2000 transfection reagent (Invitrogen, Carlsbad, California) according to the manufacturer's protocol. After a 24 h incubation period at 37 °C, the cells were incubated with fresh phenol-red free serum-free RPMI 1640 medium and treated with DHT, ethanol vehicle and/or the specified compounds in triplicate. After a 24 h treatment period the cells were washed twice with ice-cold DPBS and assayed using the Dual Luciferase kit (Promega) according to the manufacturer's protocol. Briefly, cells were lysed with 100 μl of luciferase lysing buffer, collected in a microcentrifuge tube, and pelleted by centrifugation. Supernatants (100 μl aliquots) were transferred to corresponding wells of opaque 96-well multiwell plates. Luciferin was added to each well, and the light produced during the luciferase reaction was measured in a Victor 1420 scanning multi-well spectrophotometer (Wallac, Inc., Gaithersburg, MD). After measurement, Stop and Glo reagent (Promega) was added to quench the firefly luciferase signal and initiate the *Renilla* luciferase luminescence. *Renilla* luciferase luminescence was also measured in the Victor 1420. The results are presented as the fold induction, that is, the relative luciferase activity of the treated cells divided by that of the control, normalized to that of the *Renilla* [42].

### FACS analysis

Cells were harvested by trypsinization and then ﬁxed with 70% ethanol for 24 h at 4 °C. Fixed cells were stained in 1 ml of propidium iodide solution (0.05% NP-40, 50 mg per ml propidium iodide, and 10 mg per ml RNase A) for at least 2 h at 4 °C. Stained cells were analyzed with a ﬂow cytometer using FlowJo software that exploits Watson algorithm to find out peak and S-phase populations from a univariate distribution curve.

### Cell death assessment

Apoptosis was evaluated in PCa cells (LNCaP, C4-2B and 22Rv1) by acridine orange/ethidium bromide dual staining. Briefly, cells were seeded in 12-well plate at seeding densities of 1 × 10^5^ cells and then treated with 5 μM of indicated compounds for 24 h. Subsequently cells were washed once with phosphate buffered saline and incubated with 100 μl of 1:1 mixture of acridine orange and ethidium bromide (4 μg/ml) for 30 min. Following this, cells were immediately washed with PBS and analyzed using Nikon TE2000 fluorescence microscope. Cytoplasmic histone-associated DNA fragments were quantified by using the Cell Death Detection ELISAPLUS kit (Roche Applied Science, Indianapolis, IN) according to the manufacturer's instructions. Brieﬂy, ﬂoating and attached cells were collected and homogenized in 400 μL of incubation buffer. The wells were coated with antihistone antibodies and incubated with the lysates, horseradish peroxidase–conjugated anti-DNA antibodies, and the substrate, in that sequence. Absorbance was measured at 405 nm.

### Wound healing migration assay

For wound healing assay highly metastatic PC-3 cells were plated in a 24 well plate at a seeding density of 5 × 10^5^ cells/ well and allowed to form a confluent monolayer for 24 h. Cells were made dormant by pretreating with 0.5 μmol/L mitomycin C for 2 h to ensure that wounds are filled due to cell migration and not by cell proliferation. Subsequently, the monolayer was scratched with a pipette tip, washed with media to remove floating cells, and photographed (time 0 h). Cells were then treated with indicated compounds (5 μM) and the experiment was terminated as soon as wound was completely filled in vehicle treated controls. Cells were then photographed again using Nikon TE2000 microscope at three randomly selected sites per well [43].

### Boyden chamber invasion assay

The invasion assay in PC-3 cells was performed using Matrigel (BD Biosciences, Bedford, MA, USA)-coated transwell cell culture chambers (8 μm pore size, EMD Millipore, Temecula, CA, USA) as described previously (43). Briefly, PC-3 cells (5 × 10^4^ cells/well) were cultured in the upper chamber of the transwell insert for 24 h in serum-free RPMI-1640 medium. The cells were then treated with 5 μM of indicated compounds for 24 h. RPMI-1640 medium containing 10% FBS was placed in the lower chamber. At the end of incubation, the top surface of the non-migrated cells were scraped gently with cotton swabs and the cells on the lower surface of the membrane (migrated cells) were fixed for 15 min with cold methanol and stained with crystal violet. Cells that had migrated to the bottom of the membrane were visualized and counted using an inverted microscope. For each replicate (n = 3), cells in three randomly selected fields were counted and averaged.

### Western blotting and antibodies

For western blotting, cells were lysed in modiﬁed RIPA lysis buffer (Sigma-Aldrich, St Louis, MO, USA) supplemented with a protease inhibitor mix (Thermo Scientiﬁc, Rockford, IL, USA). Unless otherwise described, 30 μg protein was resolved by SDS–polyacrylamide gel electrophoresis, transferred, and immunoblotted with using the following antibodies: AR, Bax, caspase-3, CHIP, cyclin B, cyclin D1, E-cadherin, eIF4E, MNK1, MDM2, N-cadherin, cleaved PARP, peIF4E^ser209^, PSA procured from Cell Signaling Technology, Danvers, MA, USA; anti-MNK2 was purchased from Sigma-Aldrich, St. Louis, MO, USA; and normal rabbit IgG, cyclin D1, E-cadherin, and Ub was from Santa Cruz Biotechnology, CA, USA (19).

The dose of NRs used in the present study was chosen based on the dose dependent experiment that was initially performed (data not shown). Our results revealed that NRs significantly modulated the expression of proteins analyzed at dose starting at 5 μM with maximal effect at 20 μM. Hence 20 μM concentration was chosen for performing most of the analysis. While no significant difference was observed at 5 and 20 μM concentration with NRs in cell based assays, 5 μM was uniformly chosen to perform cell based analysis.

### Gene silencing by siRNA

For siRNA transfection, 2 × 10^5^ cells were seeded in 6 cm dish for 24 h in culture medium. The cells were then transfected with 100 nM of Mnk1/AR and non-targeting siRNAs (purchased from Ambion) for 18 h using Lipofectamine® 2000 Transfection reagent (Invitrogen). Protein silencing was confirmed by immunoblot analysis. For cell growth assay experiments, transfection complex were removed after 18 h, cells were washed twice with phosphate-buffered saline and replaced with growth medium. Twenty-four hour later drug was added and harvested after 72 h. For transfection with MDM2 or CHIP siRNA, LNCaP cells were transfected with 100 nM of MDM2 or CHIP siRNA for 18 h, transfection complexes were washed off and replaced with phenol free media for 24 h. Cells were then treated with 20 μM of VNLG-152 for an additional 24 h before cell lysis by RIPA lysis buffer [44,45].

### Immunoprecipitation and ubiquitination assay

LNCaP cells were treated with VNLG-152 (20 μM) and MG-132 (5 μM) and combination thereof for 24 h, harvested and lysed in modiﬁed RIPA buffer. MG-132 was added 8 h prior to the VNLG-152. Ubiquitinated proteins were immunoprecipitated with 20 ml of protein A/G sepharose beads (Santa Cruz Biotechnology, CA, USA) for 45 min and centrifuged at 13,300 rpm for 1 min. Supernatants were then incubated with 1 μg of polyclonal antibody per 500 μg of total protein in immunoprecipitate. Protein lysate-antibody complex were rotated for 12 h at 4 °C and beads added for an additional 1 h. Complexes were centrifuged at 13,300 rpm for 1 min, and the supernatant was discarded. Beads were subsequently washed with 3X IP/lysis buffer and re-suspended in 2X SDS sample loading buffer and boiled at 99 °C for 5 min. Samples were then resolved by SDS–PAGE, and immunoblotted for ubiquitin after stripping of the membrane for AR/MNK [19].

### Statistical analysis

All experiments were carried out in at least triplicates and are expressed as mean ± S.E. where applicable. Treatments were compared to controls using the Student's t-test with either GraphPad Prism or Sigma Plot. Differences between groups were considered statistically significant at P < 0.05.

## SUPPLEMENTARY MATERIAL, FIGURES



## References

[1] Siegel R, Naishadham D, Jemal A (2013). Cancer statistics, 2013. CA Cancer J Clin.

[2] Chen CD, Welsbie DS, Tran C, Baek SH, Chen R, Vessella R, Rosenfeld MG, Sawyers CL (2004). Molecular determinants of resistance to antiandrogen therapy. Nat Med.

[3] Yuan X, Cai C, Chen S, Chen S, Yu Z, Balk SP (2014). Androgen receptor functions in castration-resistant prostate cancer and mechanisms of resistance to new agents targeting the androgen axis. Oncogene.

[4] Wendel HG, Silva RL, Malina A, Mills JR, Zhu H, Ueda T, Watanabe-Fukunaga R, Fukunaga R, Teruya-Feldstein J, Pelletier J, Lowe SW (2007). Dissecting eIF4E action in tumorigenesis. Genes Dev.

[5] Topisirovic I, Ruiz-Gutierrez M, Borden KL (2004). Phosphorylation of the eukaryotic translation initiation factor eIF4E contributes to its transformation and mRNA transport activities. Cancer Res.

[6] Lapointe J, Li C, Higgins JP, van de Rijn M, Bair E, Montgomery K, Ferrari M, Egevad L, Rayford W, Bergerheim U, Ekman P, DeMarzo AM, Tibshirani R (2004). Gene expression profiling identifies clinically relevant subtypes of prostate cancer. Proc Natl Acad Sci USA.

[7] Tomlins SA, Mehra R, Rhodes DR, Cao X, Wang L, Dhanasekaran SM, Kalyana-Sundaram S, Wei JT, Rubin MA, Pienta KJ, Shah RB, Chinnaiyan AM (2007). Integrative molecular concept modeling of prostate cancer progression. Nat Genet.

[8] Varambally S, Yu J, Laxman B, Rhodes DR, Mehra R, Tomlins SA, Shah RB, Chandran U, Monzon FA, Becich MJ, Wei JT, Pienta KJ, Ghosh D (2005). Integrative genomic and proteomic analysis of prostate cancer reveals signatures of metastatic progression. Cancer Cell.

[9] Furic L, Rong L, Larsson O, Koumakpayi IH, Yoshida K, Brueschke A, Petroulakis E, Robichaud N, Pollak M, Gaboury LA, Pandolfi PP, Saad F, Sonenberg N (2010). eIF4E phosphorylation promotes tumorigenesis and is associated with prostate cancer progression. Proc Natl Acad Sci USA.

[10] Graff JR, Konicek BW, Lynch RL, Dumstorf CA, Dowless MS, McNulty AM, Parsons SH, Brail LH, Colligan BM, Koop JW, Hurst BM, Deddens JA, Neubauer BL (2009). eIF4E activation is commonly elevated in advanced human prostate cancers and significantly related to reduced patient survival. Cancer Res.

[11] Tasseff R, Nayak S, Salim S, Kaushik P, Rizvi N, Varner JD (2010). Analysis of the molecular networks in androgen dependent and independent prostate cancer revealed fragile and robust subsystems. PLoS One.

[12] Gediya LK, Belosay A, Khandelwal A, Purushottamachar P, Njar VC (2008). Improved synthesis of histone deacetylase inhibitors (HDIs) (MS-275 and CI-994) and inhibitory effects of HDIs alone or in combination with RAMBAs or retinoids on growth of human LNCaP prostate cancer cells and tumor xenografts. Bioorg Med Chem.

[13] Huynh CK, Brodie AM, Njar VC (2006). Inhibitory effects of retinoic acid metabolism blocking agents (RAMBAs) on the growth of human prostate cancer cells and LNCaP prostate tumour xenografts in SCID mice. Br J Cancer.

[14] Patel JB, Huynh CK, Handratta VD, Gediya LK, Brodie AM, Goloubeva OG, Clement OO, Nanne IP, Soprano DR, Njar VC (2004). Novel retinoic acid metabolism blocking agents endowed with multiple biological activities are efficient growth inhibitors of human breast and prostate cancer cells *in vitro* and a human breast tumor xenograft in nude mice. J Med Chem.

[15] Belosay A, Brodie AM, Njar VC (2006). Effects of novel retinoic acid metabolism blocking agent (VN/14-1) on letrozole-insensitive breast cancer cells. Cancer Res.

[16] Njar VC, Gediya L, Purushottamachar P, Chopra P, Vasaitis TS, Khandelwal A, Mehta J, Huynh C, Belosay A, Patel J (2006). Retinoic acid metabolism blocking agents (RAMBAs) for treatment of cancer and dermatological diseases. Bioorg Med Chem.

[17] Patel JB, Khandelwal A, Chopra P, Handratta VD, Njar VC (2007). Murine toxicology and pharmacokinetics of novel retinoic acid metabolism blocking agents. Cancer Chemother Pharmacol.

[18] Patel JB, Mehta J, Belosay A, Sabnis G, Khandelwal A, Brodie AM, Soprano DR, Njar VC (2007). Novel retinoic acid metabolism blocking agents have potent inhibitory activities on human breast cancer cells and tumour growth. Br J Cancer.

[19] Ramalingam S, Gediya L, Kwegyir-Afful AK, Ramamurthy VP, Purushottamachar P, Mbatia H, Njar VC (2014). First MNKs degrading agents block phosphorylation of eIF4E, induce apoptosis, inhibit cell growth, migration and invasion in triple negative and Her2-overexpressing breast cancer cell lines. Oncotarget.

[20] Lonergan PE, Tindall DJ (2011). Androgen receptor signaling in prostate cancer development and progression. J Carcinog.

[21] Vidya Priyadarsini R, Senthil Murugan R, Maitreyi S, Ramalingam K, Karunagaran D, Nagini S (2010). The flavonoid quercetin induces cell cycle arrest and mitochondria-mediated apoptosis in human cervical cancer (HeLa) cells through p53 induction and NF-κB inhibition. Eur J Pharmacol.

[22] Deep G, Gangar SC, Agarwal C, Agarwal R (2011). Role of E-cadherin in antimigratory and antiinvasive efficacy of silibinin in prostate cancer cells. Cancer Prev Res.

[23] Sarkar S, Brautigan DL, Parsons SJ, Larner JM (2014). Androgen receptor degradation by the E3 ligase CHIP modulates mitotic arrest in prostate cancer cells. Oncogene.

[24] Bianchini A, Loiarro M, Bielli P, Busà R, Paronetto MP, Loreni F, Geremia R, Sette C (2008). Phosphorylation of eIF4E by MNKs supports protein synthesis, cell cycle progression and proliferation in prostate cancer cells. Carcinogenesis.

[25] Yu D, Scott C, Jia WW, De Benedetti A, Williams BJ, Fazli L, Wen Y, Gleave M, Nelson C, Rennie PS (2006). Targeting and killing of prostate cancer cells using lentiviral constructs containing a sequence recognized by translation factor eIF4E and a prostate-specific promoter. Cancer Gene Ther.

[26] Watson PA, Chen YF, Balbas MD, Wongvipat J, Socci ND, Viale A, Kim K, Sawyers CL (2010). Constitutively active androgen receptor splice variants expressed in castration-resistant prostate cancer require full-length androgen receptor. Proc Natl Acad Sci USA.

[27] Gaughan L, Logan IR, Neal DE, Robson CN (2005). Regulation of androgen receptor and histone deacetylase 1 by Mdm2-mediated ubiquitylation. Nucleic Acids Res.

[28] Deep G, Oberlies NH, Kroll DJ, Agarwal R (2008). Isosilybin B causes androgen receptor degradation in human prostate carcinoma cells via PI3K-Akt-Mdm2-mediated pathway. Oncogene.

[29] Lin HK, Wang L, Hu YC, Altuwaijri S, Chang C (2002). Phosphorylation-dependent ubiquitylation and degradation of androgen receptor by Akt require Mdm2 E3 ligase. EMBO J.

[30] Hou J, Lam F, Proud C, Wang S (2012). Targeting Mnks for cancer therapy. Oncotarget.

[31] Ruggero D, Montanaro L, Ma L, Xu W, Londei P, Cordon-Cardo C, Pandolfi PP (2004). The translation factor eIF-4E promotes tumor formation and cooperates with c-Myc in lymphomagenesis. Nat Med.

[32] Lim S, Saw TY, Zhang M, Janes MR, Nacro K, Hill J, Lim AQ, Chang CT, Fruman DA, Rizzieri DA, Tan SY, Fan H, Chuah CT (2013). Targeting of the MNK-eIF4E axis in blast crisis chronic myeloid leukemia inhibits leukemia stem cell function. Proc Natl Acad Sci USA.

[33] Wheater MJ, Johnson PW, Blaydes JP (2010). The role of MNK proteins and eIF4E phosphorylation in breast cancer cell proliferation and survival. Cancer Biol Ther.

[34] Fan S, Ramalingam SS, Kauh J, Xu Z, Khuri FR, Sun SY (2009). Phosphorylated eukaryotic translation initiation factor 4 (eIF4E) is elevated in human cancer tissues. Cancer Biol Ther.

[35] Ueda T, Sasaki M, Elia AJ, Chio II, Hamada K, Fukunaga R, Mak TW (2010). Combined deficiency for MAP kinase-interacting kinase 1 and 2 (Mnk1 and Mnk2) delays tumor development. Proc Natl Acad Sci USA.

[36] Balk SP, Knudsen KE AR (2008). the cell cycle, and prostate cancer. Nucl Recept Signal.

[37] Haag P, Bektic J, Bartsch G, Klocker H, Eder IE (2005). Androgen receptor down regulation by small interference RNA induces cell growth inhibition in androgen sensitive as well as in androgen independent prostate cancer cells. J Steroid Biochem Mol Biol.

[38] Shrotriya S, Gagan D, Ramasamy K, Raina K, Barbakadze V, Merlani M, Gogilashvili L, Amiranashvili L, Mulkijanyan K, Papadopoulos K, Agarwal C, Agarwal R (2012). Poly(3-(3, 4-dihydroxyphenyl) glyceric acid) from Comfrey exerts anti-cancer efficacy against human prostate cancer via targeting androgen receptor, cell cycle arrest and apoptosis. Carcinogenesis.

[39] Pulukuri SM, Gondi CS, Lakka SS, Jutla A, Estes N, Gujrati M, Rao JS (2005). RNA interference-directed knockdown of urokinase plasminogen activator and urokinase plasminogen activator receptor inhibits prostate cancer cell invasion, survival, and tumorigenicity *in vivo*. J Biol Chem.

[40] Graff JR, Boghaert ER, De Benedetti A, Tudor DL, Zimmer CC, Chan SK (1995). Reduction of translation initiation factor 4E decreases the malignancy of ras-transformed cloned rat embryo fibroblasts. Int J Cancer.

[41] Li Y, Fan S, Koo J, Yue P, Chen ZG, Owonikoko TK, Ramalingam SS, Khuri FR, Sun SY (2012). Elevated expression of eukaryotic translation initiation factor 4E is associated with proliferation, invasion and acquired resistance to erlotinib in lung cancer. Cancer Biol Ther.

[42] Purushottamachar P, Godbole AM, Gediya LK, Martin MS, Vasaitis TS, Kwegyir-Afful AK, Ramalingam S, Ates-Alagoz Z, Njar VC (2013). Systematic structure modifications of multitarget prostate cancer drug candidate galeterone to produce novel androgen receptor down-regulating agents as an approach to treatment of advanced prostate cancer. J Med Chem.

[43] Kanazawa S, Fujiwara T, Matsuzaki S, Shingaki K, Taniguchi M, Miyata S, Tohyama M, Sakai Y, Yano K, Hosokawa K, Kubo T (2010). bFGF regulates PI3-kinase-Rac1-JNK pathway and promotes fibroblast migration in wound healing. PLoS One.

[44] Grzmil M, Huber RM, Hess D, Frank S, Hynx D, Moncayo G, Klein D, Merlo A, Hemmings BA (2014). MNK1 pathway activity maintains protein synthesis in rapalog-treated gliomas. J Clin Invest.

[45] Zeng G, Apte U, Cieply B, Singh S, Monga SP (2007). siRNA-mediated beta-catenin knockdown in human hepatoma cells results in decreased growth and survival. Neoplasia.

