# Supplementary material for: Simultaneous targeting of androgen receptor (AR) and MAPK-interacting kinases (MNKs) by novel retinamides inhibits growth of human prostate cancer cell lines
Source: Oncotarget. 2014 Dec 26;6(5):3195–210. doi: 10.18632/oncotarget.3084 (PMC4413647; doi:10.18632/oncotarget.3084)
Supplement: Supplementary file 1 [file oncotarget-06-3195-s001.pdf]

# Simultaneous targeting of androgen receptor (AR) and MAPK-interacting kinases (MNKs) by novel retinamides inhibits growth of human prostate cancer cell lines

## Supplementary Material

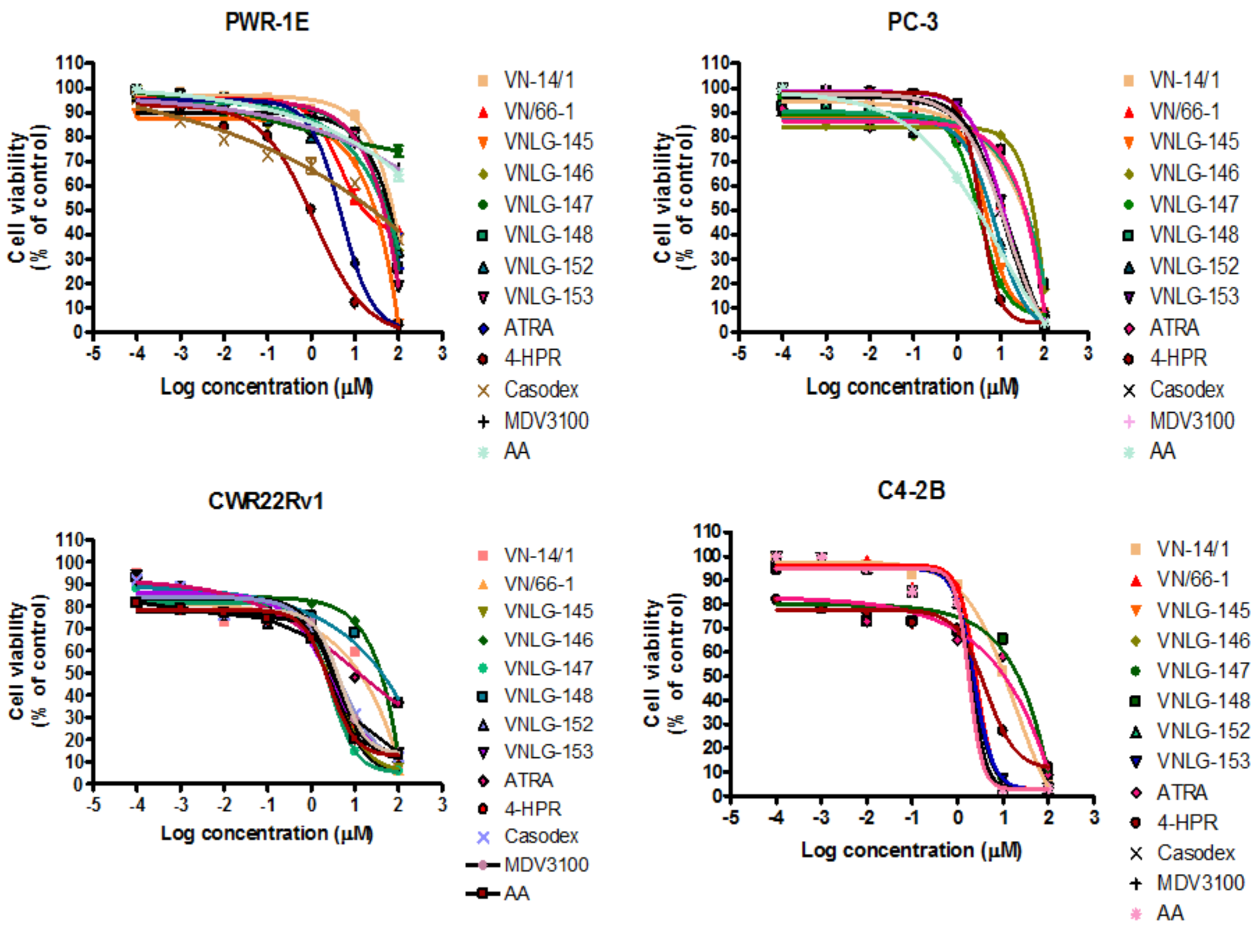


**Supplementary Figure 1: Antiproliferative potential of RRs in PWR-1E, PC-3, C4-2B and CWR22Rv1 cells.** Curves generated from an MTT assay after 7 days exposure to indicated compound. Points: mean of replicates from 3 independent experiments; bars, SE. Solid line, best-fit sigmoidal dose response (variable slope).

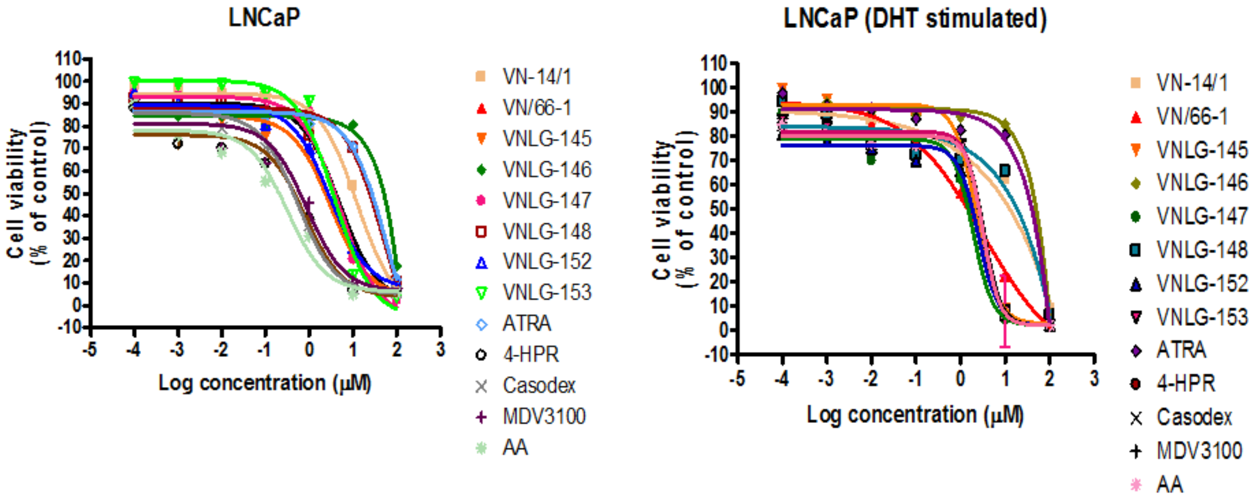


**Supplementary Figure 2: Antiproliferative potential of RRs in DHT induced and uninduced LNCaP cells.** Curves generated from an MTT assay after 7 days exposure to indicated compound. Points: mean of replicates from 3 independent experiments; bars, SE. Solid line, best-fit sigmoidal dose response (variable slope).
